# Bio-Derived Metamaterials: A Hierarchical Biomimetics-Based Evaluation System for Cross-Scale Performance in Chaozhou Woodcarving

**DOI:** 10.3390/biomimetics10100682

**Published:** 2025-10-10

**Authors:** Fan Wu, Liefeng Li, Congrong Xiao

**Affiliations:** 1 School of Design and Innovation, Guangzhou University of Software, Guangzhou 510990, China; wuf@mail.seig.edu.cn (F.W.); llf@mail.seig.edu.cn (L.L.); 2 Department of X-Cultural Studies, Kookmin University Graduate School, Kookmin University, Seoul 02707, Republic of Korea

**Keywords:** biomimetics, Chaozhou woodcarving, hierarchical structure, traditional craftsmanship, design evaluation, Analytic Hierarchy Process (AHP)

## Abstract

For centuries, artisans have resolved intricate engineering conundrums with intuitive ingenuity, bequeathing a legacy of design wisdom that remains largely untapped in contemporary biomimetics. This “anthro-creative” form of biomimicry, deeply embedded within traditional crafts such as Chaozhou woodcarving, is predominantly tacit and qualitative, which has traditionally eluded systematic interpretation. To address this, we propose the Hierarchical Biomimetics-Based Evaluation System (HBBES), a transdisciplinary framework that couples expert-defined hierarchies through the Analytic Hierarchy Process (AHP) with perceptual assessments from one hundred public evaluators via Fuzzy Comprehensive Evaluation (FCE). Applied to canonical works—including the Lobster and Crab Basket (overall score: 4.36/5.00)—the HBBES revealed a striking finding: both expert and public valuations are anchored not in structural hierarchy, but in aesthetic resonance, particularly the craft’s lifelike morphological analogy and nuanced modulation of light. Beyond offering a replicable pathway for translating artisanal intuition into operative design principles, this study proposes a culture-driven paradigm for biomimetics, bridging intangible heritage with technological innovation.

## 1. Introduction

### 1.1. The Evolution of Biomimetics: From Analogy to Abstract Principle

Biomimetics, the discipline of emulating nature’s patterns and strategies to solve human challenges, has catalyzed innovation across numerous scientific and technological fields. Its early stages were marked by direct morphological analogies, yielding pioneering solutions such as Velcro, inspired by burdock burrs, and streamlined automotive designs derived from the boxfish’s hydrodynamics [1,2,3]. As the field matured, its focus evolved from superficial imitation to the abstraction of underlying functional principles. This deeper inquiry has led to significant advances, particularly in materials science—through hierarchical structures that mimic the fracture toughness of nacre and bone—and in photonics, with metamaterials inspired by the microstructures of butterfly wings enabling unprecedented light manipulation [4,5].

This trajectory reflects an evolution from perceptual imitation to the systematic decoding of nature’s design intelligence. However, as biomimetics stands at a new threshold, its future growth hinges on addressing a critical challenge: expanding its repertoire of inspiration beyond biological exemplars and developing methodologies to systematically abstract design principles from complex, non-obvious systems [6,7,8].

### 1.2. The Next Frontier: “Culture-Driven Biomimetics” and the Artisanal Knowledge Gap

While nature has provided an inexhaustible source of inspiration, the field’s near-exclusive focus on biology has created a conceptual blind spot—overlooking an equally refined repository of structural wisdom preserved within human cultural heritage [9,10]. Traditional craftsmanship, in particular, embodies a distinctive form of “anthro-creative” biomimicry [11]. It represents a multi-generational process of empirical optimization where artisans, guided by intuitive and iterative refinement, have arrived at solutions often remarkably convergent with those shaped by natural selection [12].

Crafts such as Chaozhou woodcarving encode profound, tacit understandings of material properties, structural mechanics, and multi-objective design constraints [13,14]. Yet, this knowledge is traditionally expressed through qualitative, aesthetic, and cultural language rather than formalized analytical frameworks. This epistemological divergence creates a significant methodological gap within biomimetics: a lack of systematic tools for translating artisanal intelligence into the quantitative parameters of contemporary design and engineering. Bridging this divide is a pivotal step in the evolution of biomimetics, unlocking a vast, human-refined, and pre-validated reservoir of design strategies [15,16]. This emerging perspective, which seeks inspiration from the long history of human artifacts and aesthetic preferences, is now recognized as culture-driven biomimetics [17,18].

### 1.3. Chaozhou Woodcarving: A Precursor to Bio-Inspired Structural Design

Among the world’s diverse artisanal traditions, Chaozhou woodcarving stands as a premier model for pioneering this cultural extension of biomimetic research. Its hallmark technique—multi-layer through-carving (Tongdiao)—enables the creation of monolithic yet porous three-dimensional networks that are both structurally intricate and visually striking [19,20]. These works are not mere ornaments but active design systems that embody principles central to modern engineering: hierarchical organization for simultaneous strength and lightness, cross-scale integration, and multifunctionality marrying structural stability with sophisticated light modulation.

The existence of such an advanced design system, conceived without modern computational tools, presents a compelling “natural experiment” for testing and developing new biomimetic translation methodologies. Despite its evident relevance, a formalized framework capable of systematically deconstructing and harnessing this embedded design intelligence has remained absent.

### 1.4. Objectives and Contribution

The present study aims to extend the boundaries of biomimetics by forging a systematic intersection between engineering design and traditional craftsmanship. The primary challenge lies in the epistemological divergence between the qualitative, aesthetic-driven logic of craft and the quantitative, performance-oriented framework of modern engineering [21,22,23]. Biomimetics, by its very nature, is positioned to mediate this divide through the translation of principles derived from complex systems into functional applications.

Accordingly, the objectives of this research are threefold:

(1) To formalize the HBBES as a methodological bridge, creating a common language that translates the artistic features of Chaozhou woodcarving into quantifiable design parameters related to its structure, performance, and biomimetic qualities.
(2) To apply this biomimetic lens to a canonical work of Chaozhou woodcarving, systematically analyzing how its multi-scale architecture achieves a sophisticated balance of structural stability and aesthetic effect, thereby demonstrating the system’s analytical power.
(3) To distill the analytical findings into actionable design principles for the creation of novel bio-inspired structures and systems, explicitly linking the craft’s ingenious solutions to contemporary challenges in engineering and design.

The overarching contribution of this work is twofold. First, it introduces a methodological innovation within biomimetics by providing a structured pathway for drawing inspiration not only from biological systems but also from the rich legacy of human cultural heritage. Second, it demonstrates how a truly integrative approach—marrying the analytical rigor of engineering with the intuitive creativity of art—can yield novel design strategies and drive technological innovation, articulating a new paradigm for culture-driven biomimetic design.

## 2. Materials and Methods

**Figure 1:**
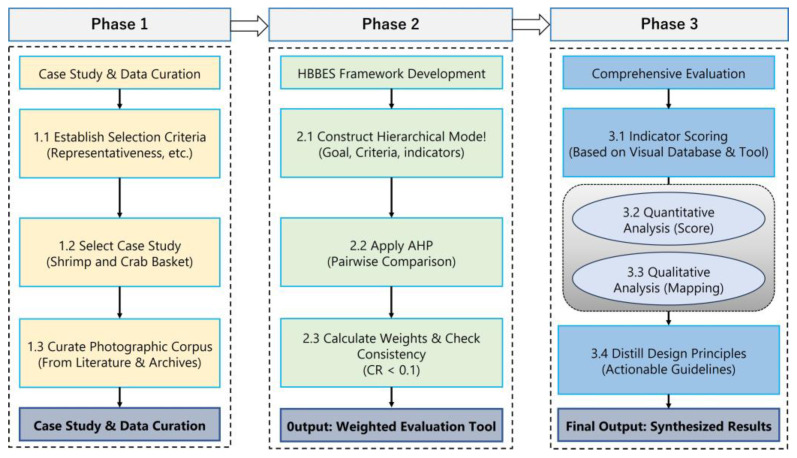
Research Roadmap.

This study adopts a multi-phase research methodology designed to systematically deconstruct and evaluate the biomimetic design intelligence embedded within Chaozhou woodcarving. The framework is grounded in a qualitative–quantitative paradigm, integrating expert-driven visual analysis with the Analytic Hierarchy Process (AHP) to transform artistic attributes into a structured and measurable evaluation system. The overall research process is schematically represented in Figure 1 and unfolds across three principal stages:

(1) Case study selection and systematic photographic documentation;
(2) Development of the Hierarchical Biomimetics-Based Evaluation System (HBBES);
(3) A comprehensive evaluation procedure.

### 2.1. Case Study Selection and Visual Data Corpus Curation

The case study was selected based on several criteria: its canonical status as a widely recognized exemplar of the craft; its structural complexity, which provided rich data for analysis; and the availability of documentation. Based on these criteria, the renowned masterpiece Lobster and Crab Basket, arguably the most celebrated work of Chaozhou woodcarving, was chosen as the subject of this investigation. Its fame ensures a wealth of existing photographic material from which to draw.

#### Curation of the Photographic Corpus

This study is based on the analysis of secondary visual data, necessitating the construction of a dedicated digital photographic corpus. The process began with an extensive, multi-channel survey encompassing academic databases (e.g., CNKI, Google Scholar), official institutional archives, high-resolution media reports, and professional image repositories. Key sources included the digital collections of national and provincial museums (e.g., The National Museum of China, Guangdong Museum), online exhibitions of specialized institutions (e.g., Chaozhou Arts and Crafts Research Institute), open resources from inheritors’ studios and local museums (e.g., Gu Liuxi Woodcarving Museum), high-quality reports from reputable media outlets (e.g., Nanfang Daily, China News Service), and licensed image banks (e.g., Visual China). The main website resource links come from http://www.czgyms.com/index.asp (accessed on 15 July 2025) (Chaozhou Arts and Crafts Research Institute).

A key challenge was the variable suitability of publicly available images for rigorous academic research. Therefore, a meticulous, multi-stage screening process with strict inclusion criteria was implemented to refine the initial pool of data:

(1) Source Reliability: Priority was given to images from sources with high curatorial or editorial standards, such as official museum collections and peer-reviewed publications, to ensure authenticity and accurate metadata.
(2) Image Quality: Only high-resolution images (>2000 pixels on the long edge) with clear focus, professional lighting, and an absence of significant digital compression artifacts or watermarks obscuring key details were included.
(3) Comprehensive Coverage: The selection aimed to create a holistic visual record for each chosen artwork, ensuring the inclusion of (a) Overall Views, (b) Sectional Views, and (c) Detailed Views where available.

**Figure 2:**
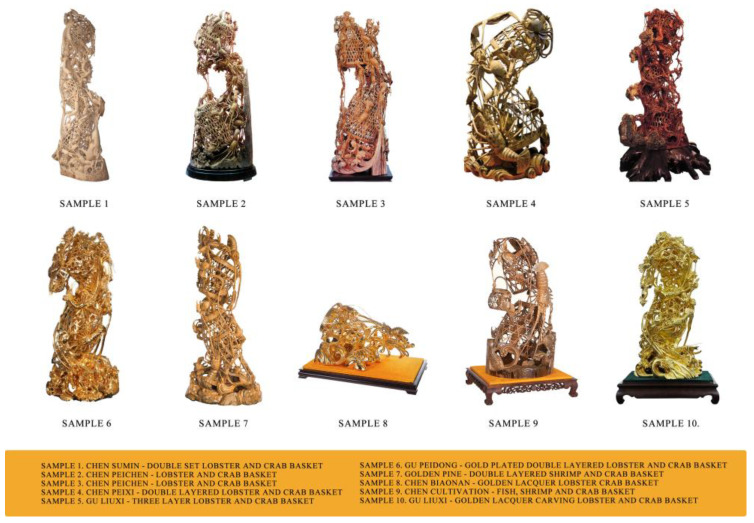
Digital photographic corpus.

Through this curation process, an initial survey of hundreds of images was systematically narrowed down to a final, targeted corpus. This corpus comprises a collection of high-quality photographs representing 10 distinct and highly representative works of the Fish, Shrimp, and Lobster and Crab Basket theme. A selection of key images from this corpus, illustrating the diversity of the chosen works, is presented in Figure 2. This final database forms the complete empirical basis for the subsequent visual analysis, and all included images were digitally collated and adjusted for uniform color balance to ensure analytical consistency.

### 2.2. Development of the Hierarchical Biomimetics-Based Evaluation System (HBBES)

The core of our methodology is the HBBES, a novel framework designed to systematically translate the qualitative features of an artisanal object into a quantitative evaluation. While the framework is tailored to Chaozhou woodcarving, its conceptual foundations are grounded in established principles from multiple disciplines. The Analytic Hierarchy Process (AHP), a well-established multi-criteria decision-making method developed by Saaty [24,25], was chosen as the mathematical engine for this framework due to its capacity to structure complex problems hierarchically and derive rational weights from expert judgments.

#### 2.2.1. Rationale and Theoretical Grounding of the Hierarchical Model

Before its construction, the dimensions of the HBBES were derived from a synthesis of relevant academic theories to ensure objectivity and validity.

Criterion 1. Structural Hierarchy (C1) is grounded in the fundamental understanding that hierarchy is a ubiquitous principle for achieving superior performance in both natural and man-made systems. Foundational research in materials science and biology has repeatedly shown that organizing structures across multiple length scales is key to achieving properties like damage tolerance and lightweight strength [26,27,28]. Our indicators (P1–P3) are therefore designed to visually assess the key manifestations of hierarchy: the number of discrete levels (P1), their spatial organization (P2), and the degree of scale separation (P3).

Criterion 2. Functional Performance (C2) follows the classic design dictum that form is intrinsically linked to function. In the context of an artistic artifact, “function” extends beyond mere mechanical performance to include aesthetic and communicative roles. Our indicators are thus chosen to capture this multi-faceted performance. Light & Shadow Modulation (P4) and Spatial Narrative Capacity (P6) are derived from principles of art and visual perception, which treat form as a medium for creating atmosphere and conveying information [29,30,31]. Concurrently, Perceived Structural Stability (P5) is based on fundamental principles of structural engineering, evaluating the intuitive logic of load paths and connections.

Criterion 3. Biomimetic Analogy (C3) is directly informed by established biomimetic theory, which distinguishes between different levels of inspiration. A critical distinction is made between mimicking an organism’s form versus its underlying principles. Our framework explicitly incorporates this distinction: Morphological Analogy (P7) corresponds to the more direct, form-based level of biomimicry, while Principle Analogy (P8) addresses the deeper, more abstract level of emulating natural strategies [32].

#### 2.2.2. Construction of the HBBES Framework

Based on this theoretical rationale, the HBBES was structured into the three-level hierarchy shown in Table 1. This structure ensures that the evaluation is not a monolithic judgment but a multi-dimensional assessment grounded in established scientific and design principles.

The relative weights of the evaluation criteria and indicators were determined using the Analytic Hierarchy Process (AHP), In this process, experts performed pairwise comparisons of all criteria and indicators using Saaty’s 9-point scale (see Table 2 for the scale explanation) to judge their relative importance. Data were gathered through a structured questionnaire survey distributed to a diverse panel of 76 stakeholders, from which 51 valid responses were collected. The expert panel included Chaozhou woodcarving artisans, de-signers, intangible cultural heritage researchers, and end-users, ensuring a comprehensive perspective. The demographic composition of the 51 respondents was well-balanced, with 50.98% male and 49.02% female participants, a majority aged between 26 and 50, and a significant contingent (approx. 20%) over 51.

To ensure the logical reliability and consistency of the expert-derived weights, the following protocol was established for processing the AHP questionnaire data:

(1) Individual Consistency Check: Each pairwise comparison matrix provided by an expert will be validated for logical consistency. The Consistency Index (CI) and Consistency Ratio (CR) will be calculated for every matrix. A CR threshold of ≤0.10 will be set as the criterion for acceptability, following established AHP methodology.
(2) Expert Screening: Experts will be included in the final analysis only if all their provided judgment matrices (for the goal and criteria levels) satisfy the CR ≤ 0.10 condition. Respondents with any inconsistent matrices will be excluded from subsequent aggregation.
(3) Aggregation of Judgments: The geometric mean of the corresponding entries from all validated, consistent matrices will be calculated to construct a single, aggregated pairwise comparison matrix for each level of the hierarchy.
(4) Final Weight Calculation: The priority vectors (weights) will then be derived from these aggregated matrices using the geometric mean method.

This rigorous protocol is designed to ensure that the final weights are derived from a pool of logically self-consistent expert judgments, thereby enhancing the robustness and validity of the HBBES framework.

### 2.3. Evaluation Procedure and Data Analysis

Following the establishment of the weighted HBBES framework, a systematic evaluation of the case study was conducted. The evaluation process involved scoring the Lobster and Crab Basket, as represented by the curated photographic corpus, against the eight indicators (P1–P8) detailed in Table 1. The evaluation procedure employs two distinct scaling systems: the AHP relies on expert pairwise comparisons using a 9-point ratio scale, while the FCE is based on public direct ratings using a 5-point Likert scale. The subsequent data analysis for both the AHP and FCE components was conducted using Microsoft Excel. All mathematical operations, including the calculation of geometric means for AHP, the construction of fuzzy membership matrices, and the defuzzification process for FCE, were executed manually within spreadsheets to ensure full transparency and traceability of the calculations.

To ensure analytical rigor and replicability, each score was guided by a detailed, pre-defined rubric that provided descriptive anchors for every level of the scale. Subsequently, these individual scores (*S_j_*) were synthesized with their corresponding local weights (*w_j_*) and criteria-level weights (*W_i_*) using a linear weighted sum model to derive a final comprehensive score for the artwork’s biomimetic design performance.

To qualitatively corroborate and visually enrich this quantitative assessment, particularly the scores related to the ‘Biomimetic Analogy’ criterion, a comparative visual analysis was performed. This involved creating a series of Comparative Visual Plates. Each plate juxtaposes a detailed photograph from the corpus with an image of its proposed biological analogue (e.g., the carved lattice vs. a microscopic view of a radiolarian skeleton), sourced from scientific literature or databases. This final step provides direct visual evidence for the biomimetic mapping and offers a more intuitive interpretation of the quantitative results.

### 2.4. Statement on Data Source

It is important to clarify that this study is a non-invasive analytical investigation that relies exclusively on secondary data. No primary data collection, such as new photography, 3D scanning, or direct physical measurement of the artifact, was conducted by the author. All visual data for the Lobster and Crab Basket were compiled from publicly accessible and reputable sources, including peer-reviewed art historical literature, official museum digital collections, and high-resolution online art archives. This approach was deliberately chosen as a respectful and appropriate method for studying a culturally significant and often physically inaccessible masterpiece. The validity of the subsequent analysis, therefore, rests on the systematic curation of the best available visual documentation and the rigorous application of the HBBES framework to this curated corpus.

## 3. Results and Analysis

This section presents the results derived from the application of the Hierarchical Biomimetics-Based Evaluation System (HBBES) to the Lobster and Crab Basket case study. The findings are presented in three parts: first, the weight distribution of the HBBES framework as determined by the AHP; second, the quantitative evaluation scores of the case study; and third, the qualitative visual analysis that corroborates these findings.

### 3.1. HBBES Weight Distribution

#### 3.1.1. Construct a Judgment Matrix

Following the protocol described in Section 2.2.2, Respondents performed pairwise comparisons for all indicators within the hierarchy using Saaty’s 1–9 fundamental scale and its reciprocals see Table 2. To synthesize the judgments of the 51 experts, the geometric mean was calculated for each entry in the comparison matrices. These aggregated matrices were then used to derive the priority vectors, which were subsequently normalized to obtain the final weights. An example of the calculation for the criteria-level judgment matrix (*M*) is shown in Table 3, with the governing matrix Equations specified in Equation (1).

The aggregated evaluation data were then used to construct the full set of pairwise comparison matrices as prescribed by the AHP methodology. This yielded the judgment matrices for the indicator level (*M*_*C*1_, *M*_*C*2_, *M*_*C*3_) and the criteria level relative to the goal (*M_O_*), which are presented in Equation (1).

(1) $$\begin{array}{l} M_{O}=\left[\begin{array}{ccc} C_{11} & C_{12} & C_{13}\\ C_{21} & C_{22} & C_{32}\\ C_{31} & C_{32} & C_{33} \end{array}\right]=\left[\begin{array}{ccc} 1 & 0.2540 & 0.2473\\ 3.9375 & 1 & 0.4286\\ 4.0435 & 2.3333 & 1 \end{array}\right]\\ M_{C1}=\left[\begin{array}{ccc} P_{11} & P_{12} & P_{13}\\ P_{21} & P_{22} & P_{32}\\ P_{31} & P_{32} & P_{33} \end{array}\right]=\left[\begin{array}{ccc} 1 & 4.3617 & 7.0400\\ 0.2293 & 1 & 3.7660\\ 0.1420 & 0.2655 & 1 \end{array}\right]\\ M_{C2}=\left[\begin{array}{ccc} P_{44} & P_{45} & P_{46}\\ P_{54} & P_{55} & P_{56}\\ P_{64} & P_{65} & P_{66} \end{array}\right]=\left[\begin{array}{ccc} 1 & 3.5833 & 6.6078\\ 0.2791 & 1 & 4.0000\\ 0.1513 & 0.2500 & 1 \end{array}\right]\\ M_{C3}=\left[\begin{array}{cc} P_{77} & P_{78}\\ P_{87} & P_{88} \end{array}\right]=\left[\begin{array}{cc} 1 & 3.8085\\ 0.2626 & 1 \end{array}\right] \end{array}$$

#### 3.1.2. Expert Screening and Aggregation Validation

Following the validation protocol established in Section 2.2.2, each of the 76 individual judgment matrices was checked for consistency. This screening process identified and excluded 25 responses that contained one or more judgment matrices with a CR value > 0.10. The subsequent analysis is therefore based on the aggregated matrices built from the remaining 51 validated expert questionnaires.

The geometric mean of the corresponding entries from these 51 consistent matrices was then calculated to construct the final aggregated pairwise comparison matrices for each level of the hierarchy (*M*_*O*_, *M*_*C*1_, *M*_*C*2_, *M*_*C*3_).

The consistency of each final aggregated matrix was also verified. The results of this check are summarized in Table 3. The CR values for all aggregated matrices are well below the 0.10 threshold, confirming that the final group judgments, derived from consistent individual inputs, are also highly consistent.

#### 3.1.3. Calculate

Using the judgment matrices from Equation (1), the priority vector (i.e., the weights) for each was calculated. This was accomplished using the geometric mean method, a standard approximation of the principal eigenvector in AHP. As specified in Equation (2), the procedure involves first calculating the geometric mean of the elements in each row of the matrix, and then normalizing the resulting vector to derive the final weights. The calculation for the criteria-level weights is exemplified in Table 3. This process [33,34] was repeated for all hierarchical levels to determine the weights for all indicators within the HBBES. The complete set of calculated weights was then compiled and sorted into a comprehensive summary, as presented in Table 4.

(2) $$W_{i}=\frac{\sqrt[{n}]{\prod_{j=1}^{n}a_{ij}}}{\sum_{i=1}^{n}\sqrt[{n}]{\prod_{j=1}^{n}a_{ij}}}(i=1,2,\ldots ,n)$$

The final weight distribution derived from the expert survey, as meticulously detailed in Table 4, offers a rich and layered narrative about the cognitive framework through which Chaozhou woodcarving is valued. It reveals a clear and definitive hierarchy of priorities that provides profound insight into the collective perception of the craft’s biomimetic value. The most striking and structurally significant finding is the overwhelming dominance of the Biomimetic Analogy (C3) criterion, which commands a substantial weight of 0.57. This result, accounting for well over half of the total evaluative weight, acts as the gravitational center for the entire evaluation, strongly indicating that in the collective view of the expert panel, the direct and perceivable similarity of the carving to natural systems is the most critical and foundational aspect of its biomimetic character. This philosophical stance is further magnified when examining the global weights of the individual indicators. Drilling down into the indicator level, Morphological Analogy (P7) emerges as the single most influential metric by a remarkable margin, possessing a global weight of 0.44. This exceptionally high value unequivocally underscores that the direct, visual imitation of natural forms and patterns—the aesthetic fidelity to a biological archetype—is perceived as the core tenet and primary achievement of the artwork’s biomimetic design.

Perhaps the most insightful, and indeed counter-intuitive, aspect of this result is how it compellingly refutes the initial hypothesis that a modern engineering-centric perspective—one that values the implicit structural ingenuity (C1)—would prevail. The data suggests a paradigmatic divergence. Stakeholders deeply embedded in this craft’s ecosystem primarily assess it not through a lens of structural optimization, but through one of aesthetic and representational fidelity to nature. This discovery can be understood by examining the real-world context of Chaozhou woodcarving. The expert panel was a holistic community of artisans, cultural inheritors, researchers, and connoisseurs. For this audience, the ultimate “performance” of the artwork is not measured in abstract metrics of load-bearing capacity or material efficiency, but in its visual and affective performance—its capacity to evoke wonder and to breathe life into inanimate material. For a master artisan, the pinnacle of their craft is achieving “verisimilitude,” capturing the spirit and form of a living creature so perfectly that the wood itself seems to transcend its own substance. The overwhelming weight assigned to Morphological Analogy (P7), therefore, is a powerful affirmation of the craft’s central cultural and artistic purpose.

In a secondary, yet still significant, position is the Functional Performance (C2) criterion, with a total weight of 0.32. An internal analysis of this criterion reinforces the main finding. Within this domain, Light & Shadow Modulation (P4) is the key contributor (Global Weight = 0.21), valued significantly more than Perceived Structural Stability (P5). This highlights a clear preference for the aesthetic function—the theatrical and dramatic potential of the design—over its more practical, engineering-related aspects. The structure’s ability to create compelling visual effects through its interaction with light is considered its most important function, again prioritizing the observer’s experience.

In stark contrast, the Structural Hierarchy (C1) criterion was deemed to be of comparatively minor significance, receiving a weight of only 0.11. This clear deprioritization is highly revealing. It suggests that the sophisticated layering and cross-scale features, while technically brilliant, are not valued as ends in themselves but are instead perceived as the invisible skeleton that supports the visible beauty. They are brilliant techniques in service of achieving the ultimate lifelike quality. The structure is ingenious precisely because it enables the aesthetic illusion. This study thus reveals a profound lesson for biomimetics: the true lesson from Chaozhou woodcarving may not be how to build a better lattice, but rather how to use complex structures to create profound beauty and the illusion of life. This compels a broader definition of bio-inspiration—one that encompasses not just nature’s ‘engineering’ but also its ‘artistry’—paving the way for a more holistic and human-centered approach to biomimetic design.

### 3.2. Quantitative Evaluation of Public Perception Using Fuzzy Comprehensive Evaluation (FCE)

To complement the expert-driven AHP weight determination, and to assess the perceptual performance of the artworks, a large-scale public evaluation was conducted. This phase aimed to validate whether the qualities deemed important by experts are effectively perceived and appreciated by a general audience. For this purpose, we adopted the Fuzzy Comprehensive Evaluation (FCE) method, which is exceptionally well-suited for capturing and quantifying the inherent ambiguity and subjectivity of public aesthetic judgment.

#### 3.2.1. Data Collection from Public Respondents

A survey was administered to 100 public respondents, recruited through online platforms and cultural forums [35]. Participants were presented with a high-resolution photographic portfolio of the ten selected case studies (Sample 1 to Sample 10). Participants were asked to rate its performance on the eight HBBES indicators (P1–P8) using a 5-point Likert scale (1 = Very Low to 5 = Very High), which corresponds to the fuzzy evaluation set V = {V1, V2, V3, V4, V5} or {Very Poor, Poor, Average, Good, Excellent} [36]. The large sample size of 100 ensures a high degree of statistical reliability for the evaluation results.

#### 3.2.2. FCE Methodology

The Fuzzy Comprehensive Evaluation (FCE) process translates the collective perceptions of the public respondents into a quantifiable score for each artwork. This involves three sequential steps:

Step 1: Construction of the Fuzzy Membership Matrix

For each artwork (sample), an individual fuzzy membership matrix *R* is constructed. This matrix quantifies the distribution of public opinions across the eight HBBES indicators (P1-P8) and the five evaluation grades. *R* is an 8 × 5 matrix, where each element *r_mn_* represents the membership degree of the *m*-th indicator to the *n*-th evaluation grade. It is calculated as the frequency of respondents assigning that grade, See Equation (3).

(3) $$r_{mn}=\frac{N_{mn}}{N}$$

where *N_mn_* is the number of respondents who assigned the **n**-th grade to the **m**-th indicator, and *N* is the total number of respondents (i.e., 100).

Step 2: Fuzzy Synthesis Operation

The comprehensive fuzzy evaluation vector *B* for each sample is obtained by synthesizing the global weight vector *W* (derived from the AHP, see Table 4 with the fuzzy membership matrix *R*. The weighted average operator model is employed for this synthesis, See Equations (4) and (5).

(4) $$B=W\circ R=(w_{1},w_{2},\ldots ,w_{8})\circ \left(\begin{array}{cccc} r_{11} & r_{12} & \ldots & r_{15}\\ r_{21} & r_{22} & \ldots & r_{25}\\ \vdots & \vdots & \ddots & \vdots \\ r_{81} & r_{82} & \ldots & r_{85} \end{array}\right)$$

(5) $$b_{n}=\sum_{m=1}^{8}w_{m}\cdot r_{mn}(n=1,2,\ldots ,5)$$

The resulting vector *B* = (b_1_, b_2_, b_3_, b_4_, b_5_) represents the overall membership degrees of the artwork to each of the five evaluation grades, taking into account the relative importance of each indicator.

Step 3: Defuzzification for Final Score

The fuzzy vector *B* is subsequently defuzzified into a single, crisp comprehensive score *S* to facilitate comparison and ranking. This is achieved by calculating the weighted sum of the evaluation grades, See Equations (6) and (7).

(6) $$S=B\cdot V^{T}=(b_{1},b_{2},b_{3},b_{4},b_{5})\cdot {\left(1,2,3,4,5\right)}^{T}$$

(7) $$S=\sum_{n=1}^{5}b_{n}\cdot V_{n}$$

where *V* = (1, 2, 3, 4, 5) is the score vector corresponding to the evaluation grades {Very Poor, Poor, Average, Good, Excellent}, respectively. The final score *S* provides a clear quantitative measure of the public’s perceived biomimetic performance for each artwork.

#### 3.2.3. Quantitative Evaluation Results

The Fuzzy Comprehensive Evaluation (FCE) was performed for all ten samples, based on the ratings of 100 public respondents. The following section first presents an illustrative, step-by-step calculation for a single case (Sample 1) to demonstrate the application of the methodology, followed by a summary and analysis of the complete results for all ten samples.

In order to provide a clear example of the FCE process, we have detailed the calculation of Example 1. According to the explanation of the method in the previous section, the detailed calculation process of Sample 1 is shown in Equation (8).

(8) $$\begin{array}{l} R_{1}=\left\{\begin{array}{ccccc} 0.04 & 0.03 & 0.14 & 0.51 & 0.28\\ 0.03 & 0.04 & 0.15 & 0.43 & 0.35\\ 0.01 & 0.02 & 0.19 & 0.52 & 0.26\\ 0.02 & 0.01 & 0.21 & 0.42 & 0.34\\ 0.01 & 0.01 & 0.25 & 0.29 & 0.44\\ 0.01 & 0.03 & 0.34 & 0.28 & 0.34\\ 0.02 & 0.03 & 0.13 & 0.42 & 0.40\\ 0.02 & 0.04 & 0.12 & 0.45 & 0.38 \end{array}\right\}\\ B_{1}=\left[\begin{array}{cccccccc} 0.07 & 0.02 & 0.01 & 0.21 & 0.08 & 0.04 & 0.44 & 0.12 \end{array}\right]\circ \left(\ldots \right)\\ B_{1}=\left[\begin{array}{ccccc} 0.0189 & 0.0252 & 0.164 & 0.411 & 0.371 \end{array}\right]\\ S_{1}=\left(0.0189*1\right)+\left(0.0252*2\right)+\left(0.164*3\right)+\left(0.411*4\right)+\left(0.371*5\right)=4.06 \end{array}$$

The Fuzzy Comprehensive Evaluation (FCE), based on the collective judgment of 100 public respondents, was applied to the ten-sample corpus. The final comprehensive scores (*Si*), which represent the public’s overall perceptual performance rating for each sample, are summarized and ranked in Table 5.

The results yield two primary findings. Firstly, the public perception of quality across these ten masterpieces is remarkably consistent and overwhelmingly positive. All samples received scores comfortably above 3.95, with an average score for the entire corpus of 4.14. The very low standard deviation of σ = 0.121 indicates that the scores are tightly clustered. This strongly suggests that, in the eyes of the public, all ten works are perceived as being of a consistently high quality, falling squarely within the “Good” to “Excellent” category. This consistency validates the selection of the corpus as a representative group of high-caliber artworks.

Secondly, despite this overall consistency, the ranking reveals a clear and meaningful hierarchy of public preference. Sample 8 distinguished itself as the definitive top-rated work, achieving the highest score of 4.36. It was followed closely by a top tier of other highly appreciated pieces, including Sample 9 (4.26) and Sample 6 (4.23). Conversely, Sample 10 (3.97) and Sample 3 (4.01), while still rated highly in absolute terms, were ranked at the lower end of this high-performing group.

The power of the HBBES framework lies in its ability to explain this hierarchy. A deeper analysis of the underlying fuzzy membership matrices (*R_i_*) indicates that the top-performing samples (e.g., Sample 4 and 9) received a significantly higher proportion of ‘5—Excellent’ ratings on the most heavily weighted indicators from the AHP analysis: P7 (Morphological Analogy) and P4 (Light & Shadow Modulation). This finding powerfully corroborates the AHP expert weighting: the qualities that experts deemed most important are indeed the primary drivers of public appreciation and the key differentiators of quality, even among masterpieces.

### 3.3. Qualitative Analysis and Biomimetic Mapping

To provide qualitative depth and visual evidence for the quantitative findings, this section presents an in-depth visual analysis of the highest-scoring work, Sample 4 (Score: 4.36). By examining its specific features through the lens of the most heavily weighted HBBES indicators, we can understand why it resonated so strongly with the public. The primary analytical tool used here is Biomimetic Mapping, where specific features of the carving are juxtaposed with their natural analogues in a series of comparative plates.

#### 3.3.1. Mastery of Morphological Analogy (P7)

**Figure 3:**
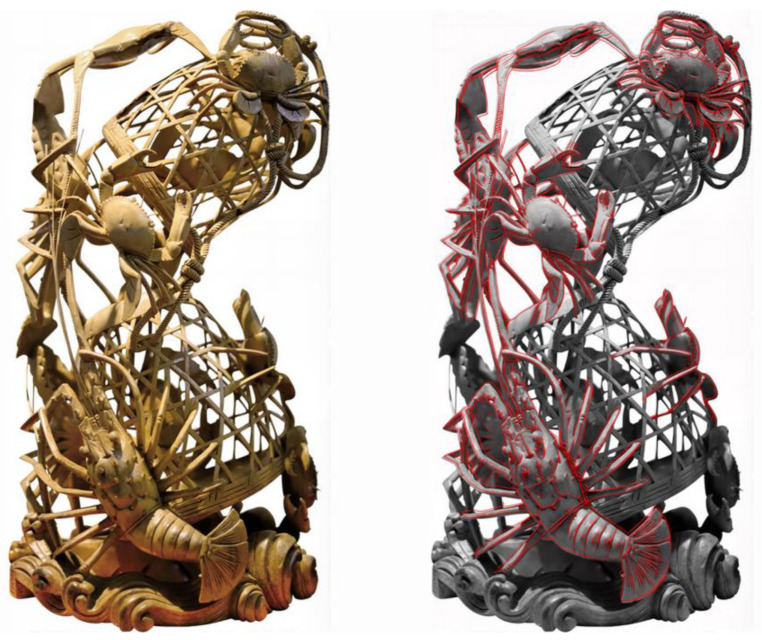
Biomimetic mapping of morphological analogy (P7).

As the most heavily weighted indicator in the entire HBBES framework (Global Weight = 0.44), Morphological Analogy was identified as the paramount criterion for success. Sample 4’s top score is largely attributable to its masterful execution in this domain. The artwork moves beyond generic representation to achieve a high degree of verisimilitude. The bodies of the shrimp and crabs are not merely suggestive but are rendered with anatomical precision; the segmentation of the exoskeletons, the delicate articulation of the legs, and the texture of the carapaces are all meticulously observed and carved. The biomimetic mapping for this indicator is best illustrated by the basket structure itself, as shown in Figure 3.

The visual analogy is particularly compelling. The latticework of the carving transcends a mere geometric grid, unfolding instead as a complex, organic, and multi-layered network of interwoven struts and voids. Morphologically, this configuration demonstrates remarkable convergence with the calcareous skeletons of colonial corals. Both manifest as lightweight, high-porosity frameworks optimized to maximize surface area while simultaneously ensuring structural stability within fluid environments. Through generations of empirical refinement, the artisans of Sample 4 independently arrived at a structural paradigm that evolutionary processes had long since perfected, thereby substantiating the public’s perception of its profound and authentic resonance with nature.

#### 3.3.2. Sophisticated Light & Shadow Modulation (P4)

**Figure 4:**
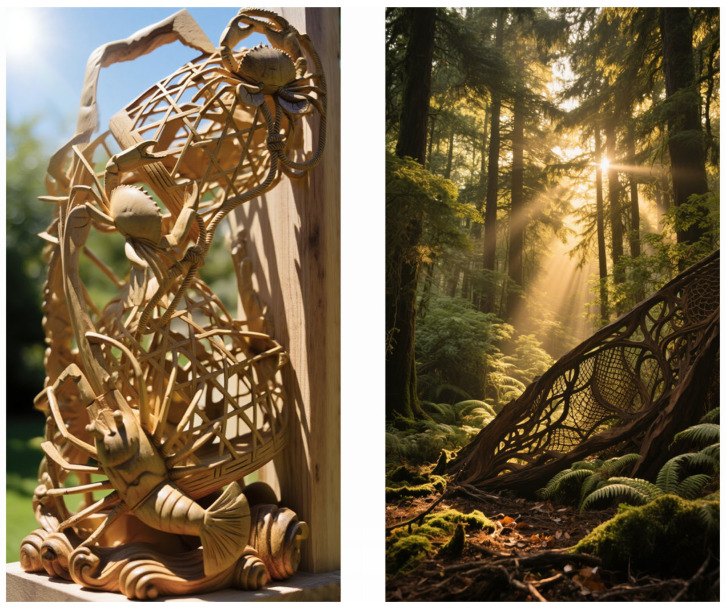
Biomimetic mapping of light & shadow modulation (P4).

The second most influential indicator, Light and Shadow Modulation (P4), pertains to the artwork’s aesthetic and functional dialogue with its surrounding environment. In this respect, Sample 4 demonstrates exceptional performance, treating the entire sculpture not as a solid mass but as a permeable, volumetric filter for light. As illumination penetrates its successive carved layers, it is refracted, occluded, and diffused, producing a dynamic chiaroscuro effect. This phenomenon extends beyond mere revelation of form; it animates the composition. The rigid edges of the wood are visually softened, the perception of spatial depth is markedly intensified, and a sense of movement is imparted to the static scene, thereby amplifying its expressive vitality and contributing significantly to the public’s high valuation. Conceptually, this principle resonates with natural optical mechanisms of light scattering and diffusion, as illustrated in Figure 4.

The underlying biomimetic principle is the use of hierarchical, semi-transparent layers to break up and modulate incident light. Just as a forest canopy creates a complex, gentle light environment on the forest floor, the layered carving transforms harsh external light into a soft, dynamic internal glow. This sophisticated understanding of light elevates the work from a mere sculpture to an interactive optical system, a key reason for its top ranking.

#### 3.3.3. The Logic of Perceived Structural Stability (P5)

**Figure 5:**
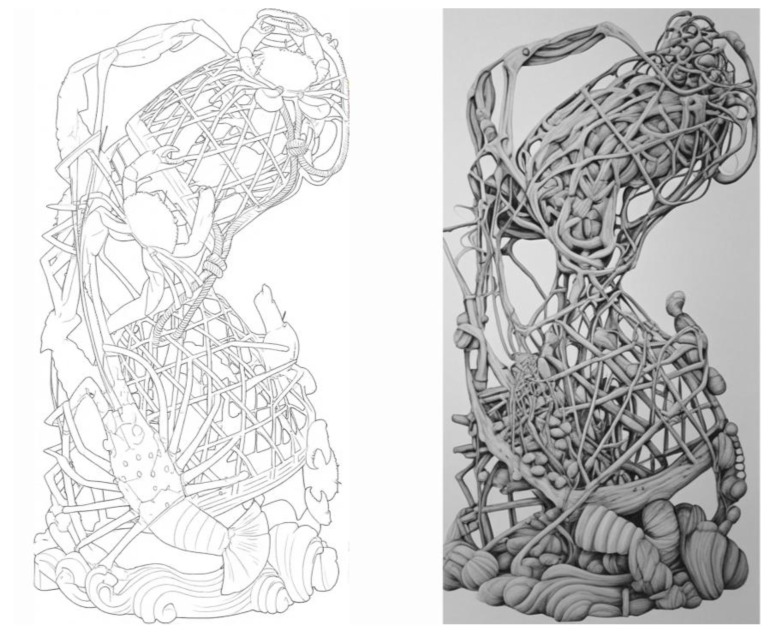
Biomimetic mapping of perceived structural stability (P5).

While less weighted than the aesthetic criteria, the public’s high scores for Sample 4 also reflect an intuitive appreciation for its structural logic. Despite its extreme porosity and delicate appearance, the work conveys a powerful sense of stability. This is achieved through subtle but critical design choices: the main frame of the basket is visibly thicker and acts as a primary load-bearing exoskeleton, while the internal elements are connected through a network of branching supports that mimic cantilevered structures, ensuring no part is isolated or fragile. This intuitive engineering finds a direct analogue in lightweight biological structures, as shown in Figure 5.

The shared principle is hierarchical branching for efficient load distribution. Trabecular bone is a natural masterpiece of lightweight design, using a lattice of struts and rods to resist multi-directional stresses while minimizing mass. Sample 4 employs the same concept, creating a visually light yet structurally sound object. The public, while not consciously analyzing the engineering, perceives the result as elegance and masterful craftsmanship, leading to a high score in this dimension.

## 4. Discussion

The wisdom of master artisans is often embodied in tacit knowledge rather than codified principles, presenting a fundamental challenge to its translation into explicit, transferable design knowledge. To address this challenge, this study introduces a novel mixed-method framework, HBBES, to systematically deconstruct the biomimetic logic embedded within Chaozhou woodcarving. By integrating expert-defined priorities via the Analytic Hierarchy Process (AHP) with public perception through Fuzzy Comprehensive Evaluation (FCE), the framework yields a multi-layered interpretation of the craft. The proposed framework advances methodological approaches in biomimetics and design theory while offering novel perspectives for the preservation and scholarly re-evaluation of intangible cultural heritage.

### 4.1. The Primacy of Analogy: Re-Framing “Performance” in a Cultural Context

A primary and counter-intuitive finding of this research is the pronounced prioritization of Biomimetic Analogy (C3) in the expert-driven AHP weighting. Our initial hypothesis, informed by a technocentric engineering perspective, posited that the craft’s structural ingenuity—its solution to achieving stability in a lightweight, porous form—would be its most valued biomimetic quality. The results compellingly refute this assumption. The craft’s expert custodians primarily assess its merit not through a lens of structural optimization, but through one of aesthetic and representational fidelity to nature.

This discovery is strongly reinforced by the public evaluation results. The FCE analysis revealed that the highest-scoring works, such as Sample 4, were precisely those that excelled in the most heavily weighted indicators. This alignment reveals a compelling congruence: the qualities most valued by experts are also those most positively perceived by the public. This suggests that the “performance” of a cultural artifact like Chaozhou woodcarving transcends mere mechanical function. Instead, its value is a measure of its visual and affective performance—its capacity to evoke wonder and an emotional connection with the viewer. This finding resonates with design theories positing that the emotional and reflective dimensions of design often create deeper user engagement than purely functional aspects. The public’s appreciation appears driven not by a conscious analysis of load paths, but by the immediate, visceral impact of lifelike representation. The sophisticated structure and light modulation are not ends in themselves, but rather techniques employed in service of this mimetic fidelity—a quality that evolutionary aesthetics suggests is a deeply ingrained and cross-culturally valued human preference [37,38].

### 4.2. Theoretical Contribution: HBBES as a Trans-Disciplinary Bridge and a New Biomimetic Paradigm

Methodologically, this work’s primary contribution is the establishment of the HBBES framework. This framework serves as a vital transdisciplinary bridge, creating a structured taxonomy and common language that enables substantive dialogue between the often-siloed domains of qualitative art history, quantitative public perception, and engineering design. By systematically translating the tacit knowledge of artisans into an explicit, hierarchical set of parameters, HBBES offers a replicable methodology to codify and analyze artisanal wisdom. This process is essential for the preservation and contemporary re-interpretation of intangible cultural heritage.

Theoretically, this study proposes a significant expansion of the inspirational sources for biomimetics. While the field has traditionally and fruitfully drawn from what has been termed the “first book” of nature, our work demonstrates that the “second book”—the rich repository of human cultural heritage—is an equally fertile yet largely untapped source of design wisdom. The strong congruence between expert judgments and public perception suggests that these crafts embody time-tested solutions that are not only structurally robust but also deeply resonant with human cognition. We therefore propose a new, complementary paradigm: culture-driven biomimetics. This approach, which derives inspiration from the long evolutionary history of human artifacts and aesthetic preferences, has the potential not only to uncover novel design solutions but also to imbue resulting technologies with deeper cultural significance and enhanced human-centric appeal.

### 4.3. Practical Implications: Actionable Principles for Contemporary Design

Beyond its theoretical implications, this research distills the analysis of Chaozhou woodcarving into several actionable principles for contemporary designers, particularly in fields where performance and human experience are inextricably linked:

Aesthetic-Driven Structuralism: The core lesson is that the structural system can be subservient to the aesthetic intent. In designing complex forms, the primary driver can be the desired visual and emotional effect (e.g., mimicking a coral’s form), with computational optimization then applied as a powerful tool to realize this vision. This flips the conventional “form follows function” mantra, famously coined by Louis Sullivan, to a more nuanced “form and function co-evolve towards an aesthetic and affective goal”.

Volumetric Light Modulation: The carvings should be understood not as inert surfaces, but as deep, permeable volumes that act as dynamic optical filters. This principle can inspire the design of building envelopes that passively regulate solar gain while creating what architects call “dappled light” atmospheres, or product casings that use layered metamaterials to create unique visual effects and illusions of depth.

High-Fidelity Bio-Morphism: The public’s strong preference for high-fidelity morphological analogy suggests that in bio-inspired design, a higher degree of realism and detail can lead to greater user appreciation and perceived value. This is a crucial consideration for designers navigating the spectrum between abstract bio-morphism and more literal, representational interpretations.

### 4.4. Limitations and Future Perspectives

While this study establishes a novel and robust framework, its limitations illuminate clear avenues for future research. First, the public evaluation was based on 2D photographic data, which cannot fully capture the three-dimensional presence of the objects. Future research could employ immersive technologies like Virtual Reality (VR) to present digital models to the public, a method shown to enhance spatial understanding and elicit more ecologically valid user responses in design evaluations [39].

Second, our analysis infers structural stability through visual perception. A logical next step would be to create high-fidelity 3D digital models of the top-performing samples and conduct Finite Element Analysis (FEA) to quantitatively simulate their structural performance under various load conditions. This would provide an empirical validation of the artisans’ intuitive engineering and could reveal optimized load-bearing strategies hidden within the art form.

Finally, the design principles extracted here could be used to generate parametric models for additive manufacturing. Fabricating and physically testing these bio-inspired prototypes would provide the ultimate validation of the craft’s engineering wisdom and demonstrate a complete “art-to-part” workflow. This would fully realize the potential of culture-driven biomimetics and contribute to the growing field of bio-inspired digital fabrication, a domain where computation, fabrication, and biology converge.

### 4.5. Generalizability of the HBBES: A Framework Beyond Chaozhou Woodcarving

While this study establishes a novel and robust framework, its limitations illuminate clear avenues for future research that extend toward sustainable design.

First, the public evaluation was based on 2D photographic stimuli, which cannot fully capture the three-dimensional presence and spatial qualities of the objects. Future research should employ immersive technologies, such as Virtual Reality (VR), to present high-fidelity digital models to participants—a method known to enhance spatial understanding and elicit more ecologically valid responses in design evaluations.

Second, our analysis infers structural stability through visual perception. A crucial next step is to conduct Finite Element Analysis (FEA) on 3D digital models of the top-performing samples. This would not only provide empirical validation of the artisans’ intuitive engineering but could also quantify the craft’s inherent material efficiency—a key principle of sustainable design. Such analysis could reveal optimized load-bearing strategies that achieve maximum structural performance with minimal material usage, offering valuable lessons for contemporary lightweight and resource-conscious engineering.

Finally, the design principles extracted herein can be translated into parametric models for additive manufacturing. This process would not only demonstrate a complete “art-to-part” workflow but would also bridge this ancient wisdom with modern sustainable manufacturing paradigms [40]. Fabricating and physically testing these bio-inspired prototypes would provide definitive validation of the craft’s engineering wisdom. By translating principles honed in a pre-industrial, resource-aware context into designs optimized for low-waste additive manufacturing, this research could yield prototypes that are not only structurally novel and aesthetically resonant but also inherently material-efficient. This would fully realize the potential of culture-driven biomimetics as a source of inspiration for a more sustainable and human-centered design ethos, contributing to the growing field where computation, fabrication, biology, and ecological principles converge [41].

## 5. Conclusions

This study set out to address a critical gap in biomimetic design: the lack of systematic methodologies to translate the tacit intelligence of traditional craftsmanship into explicit, operational design principles. In response, we developed and applied the Hierarchical Biomimetics-Based Evaluation System (HBBES), a novel transdisciplinary framework that integrates analytical rigor with perceptual assessment.

Our findings reveal a profound and somewhat counter-intuitive insight. Contrary to our initial technocentric hypothesis, the biomimetic value of Chaozhou woodcarving is judged not primarily by its hidden structural ingenuity, but by its overt aesthetic and morphological resonance with nature. Both expert-derived weights (AHP) and public perception scores (FCE) converged to show that Morphological Analogy (P7) and Light & Shadow Modulation (P4) are the most valued attributes. This indicates that the ultimate “performance” of such cultural artifacts is measured by their ability to evoke wonder and achieve verisimilitude—their capacity to harmonize functional logic with artistic illusion. The sophisticated hierarchical structure, while technically brilliant, is perceived as the enabler of this beauty, not the end itself.

The contribution of this work is twofold. Methodologically, the HBBES provides a robust and replicable framework for bridging the epistemological divide between artisanal intuition and modern engineering analysis. It offers a structured pathway to codify qualitative craft knowledge into quantitative parameters, a process vital for the preservation and contemporary re-interpretation of intangible cultural heritage. Theoretically, this study champions a significant expansion of biomimetics beyond biological inspiration. We introduce and validate a culture-driven biomimetics paradigm, positioning human cultural heritage as a rich and largely untapped source of design wisdom that is both structurally sound and deeply resonant with human cognition.

Despite these contributions, we acknowledge the study’s inherent limitations, which primarily stem from its exploratory nature and its reliance on two-dimensional photographic data. While this approach provided a non-invasive and widely accessible method for initial analysis, it necessarily constrained our ability to perform a comprehensive, quantitative assessment of the true three-dimensional structural performance—such as precise load-bearing capacity, stress distribution, and material strain. Consequently, the engineering claims made in this work are inferential, derived from visual perception and theoretical convergence with known natural principles, rather than from empirical mechanical validation.

These limitations do not diminish our findings but instead chart a clear course for future research. They underscore that this work establishes a crucial foundational framework (HBBES) upon which more advanced, technical analyses can be built. A logical and critical next step involves the creation of high-fidelity 3D digital models of these artifacts via photogrammetry or laser scanning. Such models would enable rigorous computational simulations using Finite Element Analysis (FEA) to quantitatively validate the intuitive structural genius of the artisans. Furthermore, they would facilitate perception studies in immersive Virtual Reality (VR) environments to gain deeper insights into spatial appreciation and aesthetic experience, moving beyond the constraints of 2D imagery. This proposed pathway—from cultural and qualitative evaluation to subsequent technical validation—represents a comprehensive research agenda to fully realize the potential of culture-driven biomimetics.

The design principles distilled from our analysis—Aesthetic-Driven Structuralism, Volumetric Light Modulation, and High-Fidelity Bio-Morphism—offer actionable guidance for fields like architecture, product design, and additive manufacturing. They demonstrate how artifacts long celebrated for their beauty can also serve as repositories of sophisticated design strategies with direct contemporary applicability.

Ultimately, this research affirms that traditional crafts should be viewed not as static relics, but as dynamic, transgenerational knowledge systems. By formally translating their embedded logic into applicable principles, culture-driven biomimetics opens new pathways for innovation, uniting heritage and technology in the pursuit of more human-centered, culturally nuanced, and sustainable design futures.

## Figures and Tables

**Table 1:** The hierarchical structure, indicators, and evaluation criteria of the Hierarchical Biomimetics-Based Evaluation System (HBBES).

Criteria Layer	Indicator Code	Indicator Name	Description for Visual Assessment
C1: Structural Hierarchy (The organizational complexity of the structure across scales)	P1	Perceived Visual Layers	The number of distinct, overlapping planes of carving that can be clearly identified from frontal and oblique photographs, creating foreground-background relationships.
	P2	Sense of Spatial Depth	The perceived degree of three-dimensional illusion and volumetric complexity created through compositional techniques like foreshortening, overlap, and the interplay of light and shadow within the 2D image.
	P3	Scale Contrast	The visual ratio between the largest structural elements (e.g., the main frame) and the finest carved details (e.g., an insect’s antenna), as assessed by comparing overall and detailed photographic views.
C2: Functional Performance (The effectiveness of the design in achieving visual and inferred structural goals)	P4	Light & Shadow Modulation	The structure’s capacity to actively sculpt light and cast shadows. This is assessed by the richness, contrast, complexity, and dramatic effect of the light-shadow interplay evident in the photographs.
	P5	Perceived Structural Stability	The logical and visually convincing distribution of key support points, joints, and load-bearing paths that convey a sense of stability and elegance despite the structure’s high porosity and delicate appearance.
	P6	Spatial Narrative Capacity	The efficiency with which the composition utilizes layered space to arrange multiple, complex elements to convey a coherent and dynamic scene or story within a limited volume.
C3: Biomimetic Analogy (The degree of similarity to natural systems in form and principle)	P7	Morphological Analogy	The degree of direct visual similarity between specific carved forms (e.g., meshworks, branching supports, cellular patterns) and biological analogues (e.g., corals, leaf veins, radiolarian skeletons), assessed via side-by-side image comparison.
	P8	Principle Analogy	The inferred similarity between the underlying design principles employed in the carving (e.g., porosity for lightness, branching for load distribution, hierarchy for toughness) and those found in natural biological systems.

**Table 2:** Explanation of Scale Determination.

Scale	Rating Level	Meaning
1	equally important	Indicator i is equally important as indicator j
3	Slightly important	Indicator i is slightly more important than indicator j
5	Strong and important	Indicator i is stronger and more important than indicator j
7	Strongly Important	Indicator i is stronger and more important than indicator j
9	extremely important	Indicator i is extremely more important than indicator j
2/4/6/8	median	The middle value of the two adjacent fingers mentioned above
countdown	Reverse comparison	Comparison between indicator j and indicator i, and the reciprocal of the corresponding scale

**Table 3:** Weight Calculation Details Table Using Mo Matrix as an Example.

Indicator	Judgment Matrix			Line Element Product	N th Root	*Wi*
	C1	C2	C3			
C1	1.0000	0.2540	0.2473	0.06	0.40	0.11
C2	3.9375	1.0000	0.4286	1.69	1.19	0.32
C3	4.0435	2.3333	1.0000	9.43	2.11	0.57

**Table 4:** Weight distribution for the HBBES criteria and indicators.

Criteria Layer	Weight (*W_i_*)	Indicator Code	Indicator Name	Local Weight (*w_j_*)	Global Weight (*W_i_* * *w_j_*)
C1: Structural Hierarchy	0.11	P1	Perceived Visual Layers	0.71	0.07
		P2	Sense of Spatial Depth	0.22	0.02
		P3	Scale Contrast	0.08	0.01
C2: Functional Performance	0.32	P4	Light & Shadow Modulation	0.68	0.21
		P5	Perceived Structural Stability	0.24	0.08
		P6	Spatial Narrative Capacity	0.08	0.04
C3: Biomimetic Analogy	0.57	P7	Morphological Analogy	0.79	0.44
		P8	Principle Analogy	0.21	0.12

**Table 5:** FCE Results of Public Perception Survey.

Sample ID	Final Comprehensive Score (*S_i_*)	Rank
Sample 1	4.06	8
Sample 2	4.10	6
Sample 3	4.01	9
Sample 4	4.36	1
Sample 5	4.07	7
Sample 6	4.23	3
Sample 7	4.13	5
Sample 8	4.21	4
Sample 9	4.26	2
Sample 10	3.97	10

## Data Availability

The original contributions presented in this study are included in the article. Further inquiries can be directed to the corresponding author.
